# Scavenging mitochondrial hydrogen peroxide by peroxiredoxin 3 overexpression attenuates contractile dysfunction and muscle atrophy in a murine model of accelerated sarcopenia

**DOI:** 10.1111/acel.13569

**Published:** 2022-02-24

**Authors:** Bumsoo Ahn, Rojina Ranjit, Parker Kneis, Hongyang Xu, Katarzyna M. Piekarz, Willard M. Freeman, Michael Kinter, Arlan Richardson, Qitao Ran, Susan V. Brooks, Holly Van Remmen

**Affiliations:** ^1^ Aging & Metabolism Research Program Oklahoma Medical Research Foundation Oklahoma City Oklahoma USA; ^2^ Department of Internal Medicine Wake Forest School of Medicine Winston‐Salem North Carolina USA; ^3^ Oklahoma Center for Neuroscience University of Oklahoma Health Sciences Center Oklahoma City Oklahoma USA; ^4^ Genes and Human Disease Research Program Oklahoma Medical Research Foundation Oklahoma City Oklahoma USA; ^5^ Oklahoma Nathan Shock Center for Aging Oklahoma City Oklahoma USA; ^6^ Department of Biochemistry OUHSC Oklahoma City Oklahoma USA; ^7^ Oklahoma City VA Medical Center Oklahoma City Oklahoma USA; ^8^ Department of Cell Systems & Anatomy UT Health San Antonio San Antonio Texas USA; ^9^ Department of Molecular and Integrative Physiology University of Michigan Ann Arbor Michigan USA; ^10^ Department of Physiology OUHSC Oklahoma City Oklahoma USA

**Keywords:** aging, hydrogen peroxide, mitochondria, peroxiredoxin3, sarcopenia

## Abstract

Age‐related muscle atrophy and weakness, or sarcopenia, are significant contributors to compromised health and quality of life in the elderly. While the mechanisms driving this pathology are not fully defined, reactive oxygen species, neuromuscular junction (NMJ) disruption, and loss of innervation are important risk factors. The goal of this study is to determine the impact of mitochondrial hydrogen peroxide on neurogenic atrophy and contractile dysfunction. Mice with muscle‐specific overexpression of the mitochondrial H_2_O_2_ scavenger peroxiredoxin3 (mPRDX3) were crossed to Sod1KO mice, an established mouse model of sarcopenia, to determine whether reduced mitochondrial H_2_O_2_ can prevent or delay the redox‐dependent sarcopenia. Basal rates of H_2_O_2_ generation were elevated in isolated muscle mitochondria from Sod1KO, but normalized by mPRDX3 overexpression. The mPRDX3 overexpression prevented the declines in maximum mitochondrial oxygen consumption rate and calcium retention capacity in Sod1KO. Muscle atrophy in Sod1KO was mitigated by ~20% by mPRDX3 overexpression, which was associated with an increase in myofiber cross‐sectional area. With direct muscle stimulation, maximum isometric specific force was reduced by ~20% in Sod1KO mice, and mPRDX3 overexpression preserved specific force at wild‐type levels. The force deficit with nerve stimulation was exacerbated in Sod1KO compared to direct muscle stimulation, suggesting NMJ disruption in Sod1KO. Notably, this defect was not resolved by overexpression of mPRDX3. Our findings demonstrate that muscle‐specific PRDX3 overexpression reduces mitochondrial H_2_O_2_ generation, improves mitochondrial function, and mitigates loss of muscle quantity and quality, despite persisting NMJ impairment in a murine model of redox‐dependent sarcopenia.

## Graphical abstract

Compared to innervating wildtype muscle (Top), redox‐dependent sarcopenia is accompanied by increased neuromuscular junction disruption and hydrogen peroxide from the mitochondria (Middle). Scavenging mitochondrial hydrogen peroxide protects the muscle from contractile dysfunction and atrophy independent of NMJ disruption in a mouse model of redox dependent sarcopenia (Bottom).

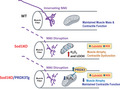

## 1 INTRODUCTION

The progressive loss of muscle mass and strength with age, termed sarcopenia, contributes to a limited capacity for daily activities in the elderly, compromising quality of life and health span. Although sarcopenia is a universal problem, the underlying mechanisms still remain elusive. Our previous studies have revealed that one of the key contributing factors is elevated oxidative stress. Excess reactive oxygen species and oxidative modifications have been linked to a number of phenotypes associated with sarcopenia including impaired contractile function and activation of proteases leading to degradation of muscle proteins (Andrade et al., 1998; Plant et al., 2001). Superoxide anion released by the mitochondrial electron transport system is converted to radicals and non‐radical oxidants, including hydroxyl radical, peroxynitrite, and peroxides (Muller et al., 2008). Because superoxide and its derivatives generate oxidative damage to cellular lipids, proteins, and DNA (Powers & Jackson, 2008), regulation of superoxide is the first and primary line of defense in response to oxygen‐derived damage.

We and others have previously demonstrated a mouse model of redox‐dependent sarcopenia, mice lacking superoxide dismutase 1 (Sod1), superoxide scavenger primarily localized in cytoplasm and intermembrane space (Jang et al., 2010; Jang & Van Remmen, 2011; Muller et al., 2006). The Sod1KO mice recapitulate key phenotypes seen in sarcopenia exhibiting high levels of oxidative stress, mitochondrial defects, and neuromuscular junction (NMJ) disruption that are typically seen in aged humans and animals (Lexell & Downham, 1991; Tomonaga, 1977). The mice, however, do not exhibit significant behavioral or other physiological alterations accompanied by advanced age, including inactivity, decreases in food consumption, or hormonal changes (Jang et al., 2010; Muller et al., 2006), serving as a model to investigate the mechanistic role of oxidative stress in sarcopenia.

Major reactive oxygen species (ROS) and oxidants involved in cellular oxidative modifications and damage are superoxide and hydrogen peroxide. Specifically, excess hydrogen peroxide is shown to impair contractile properties of skeletal muscle. Incubating isolated skeletal muscle with hydrogen peroxide resulted in significant force deficit, which was reversed by a reducing agent, dithiothreitol (Andrade et al., 1998; Plant et al., 2001). While multiple sources are involved in the production of hydrogen peroxide (Powers & Jackson, 2008), mitochondria are one of the primary sources in multiple pathological conditions, including chronic inflammatory diseases and aging (Mansouri et al., 2006). However, the significance of scavenging mitochondrial hydrogen peroxide as therapeutic target in sarcopenia is still unclear. Peroxiredoxins are one of the primary antioxidant enzymes that scavenge hydrogen peroxide along with catalase and glutathione peroxidase. Specifically, peroxiredoxin 3 (Prdx3) is shown to be exclusively expressed in mitochondrial matrix (Chen et al., 2008; Lee et al., 2014), unlike the other two enzymes that scavenge hydrogen peroxide.

Prdx3 is a critical regulator of mitochondrial hydrogen peroxide, presumably scavenging ~90% of the hydrogen peroxide generated in this compartment (Chang et al., 2004; Cox et al., 2009). The whole body Prdx3 knockout mouse model shows impaired mitochondrial homeostasis, an early onset of fatigue (Lee et al., 2014) and decreased force generation in skeletal muscle (Zhang et al., 2016). While these studies support the key role of Prdx3 in mitochondrial function and during aerobic exercise, here, we asked whether overexpression of Prdx3 can protect muscle mass and function induced by oxidative stress. Muscle‐specific overexpression of Prdx3 in Sod1KO mice allows us to directly investigate the therapeutic potential of scavenging the mitochondrial hydrogen peroxide in redox‐dependent sarcopenia. We also asked whether preventing muscle‐derived hydrogen peroxide can attenuate disruption of NMJ and reduce loss of innervation. Such a retrograde impact of skeletal muscle fibers on motor neurons has been postulated (Chakkalakal et al., 2010), but the significance of skeletal muscle‐derived hydrogen peroxide in neurogenic atrophy and weakness remains as a gap in the literature. The results from this study can help us narrow our focus for future drug interventions that can mitigate sarcopenia.

## 2 RESULTS

**FIGURE 1:**
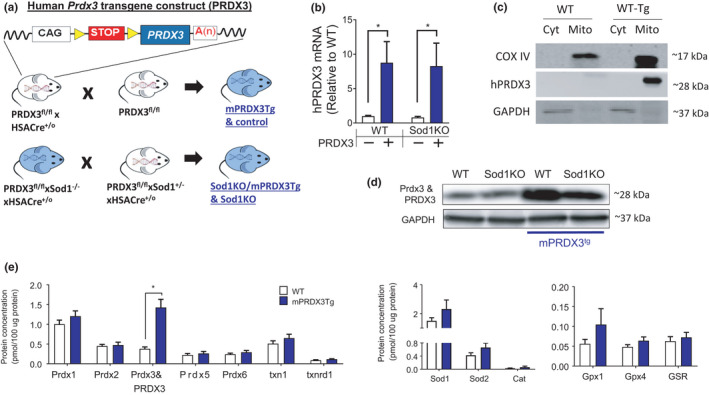
Cre‐Lox approach increases hPRDX3 expression in skeletal muscle mitochondria without upregulation of other antioxidant enzymes. (a) A schematic of peroxiredoxin3 human transgene construct (PRDX3) demonstrates a flanked STOP codon by LoxP sites. The PRDX3^fl/lf^ mice were bred to mice containing Cre recombinase driven by Human Skeletal Actin (HSA)‐Cre promotor to induce constitutive expression of PRDX3 in skeletal muscle. (b) The mRNA expression of human PRDX3. *n* = 6. Data were analyzed using ordinary two‐way ANOVA with Tukey post hoc tests. (c) Representative immunoblot images showing PRDX3 expression of mitochondrial and cytosolic fractions. Human PRDX3 antibody was used for the assay. (d) Representative immunoblots demonstrating human PRDX3 and mouse Prdx3 protein expression in WT, Sod1KO, WT‐PRDX3tg, and Sod1KO‐PRDX3tg from whole muscle homogenate. The antibody detects both human PRDX3 and mouse Prdx3 proteins. (e) Protein expression of key antioxidant enzymes using a targeted proteomics approach in muscle homogenates. *n* = 4. Student *t*‐tests were used comparing means between WT and mPRDX3 groups. Values are shown mean ± SEM. *p < 0.05. Prdx, peroxiredoxin; m Prdx3, mouse Prdx3; PRDX3, human PRDX3; txn, thioredoxin; txnrd, thioredoxin reductase; Sod, superoxide dismutase; Cat, catalase; Gpx, glutathione peroxidase; GSR, glutathione reductase

To test whether scavenging hydrogen peroxide from skeletal muscle mitochondria can rescue phenotypes associated with sarcopenia, we generated mice that constitutively express human peroxiredoxin 3 in skeletal muscle (mPRDX3Tg mice). Figure 1a depicts a schematic of the construct used to generate these mice using STOP codon flanked by LoxP sites upstream of PRDX3 transgene. The floxed mice were bred to mice harboring the HSA promotor that drives the release of the STOP codon inducing constitutive expression of the PRDX3 transgene in skeletal muscle. We found a several fold induction of human PRDX3 gene in whole muscle homogenate (Figure 1b). Likewise, protein expression of PRDX3 was upregulated in mitochondrial fraction of the skeletal muscle of the wild‐type and Sod1KO mice (Figure 1c,d). These data show that the nuclear gene‐encoded PRDX3 transgene is translated and successfully transported to the mitochondria. We also determined protein expression of PRDX3 in non‐skeletal muscle tissue homogenates in the mPRDX3Tg mice, and the protein expression was unchanged (Figure S1A). Using a targeted proteomics approach, we determined the protein expression of a panel of antioxidant enzymes in muscle homogenates. These data revealed that overexpression of PRDX3 in the transgenic mice is specific to PRDX3 protein without an up‐ or downregulation of other antioxidant enzymes in muscle (Figure 1e). Collectively, our Cre‐Lox approach induced PRDX3 upregulation in skeletal muscle mitochondria without a significant off‐target effects or compensatory increases in other antioxidant enzymes.

**FIGURE 2:**
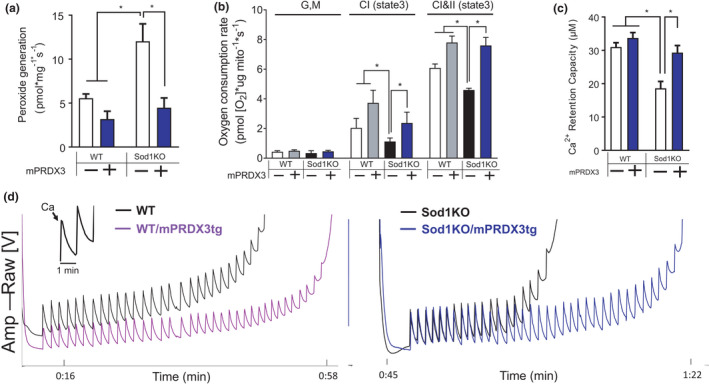
Impaired mitochondrial function in Sod1KO mice is prevented in the Sod1KO/mPRDX3Tg mice. (a) Rate of hydrogen peroxide generation in basal state without substrates or inhibitors (i.e. State 1). Significant main effects by Sod1 and mPRDX3. *n* = 4–7. (b) Oxygen consumption rate (OCR) with sequential addition of substrates for complex I and II using isolated skeletal muscle mitochondria. *n* = 4–7. (c) Calcium retention capacity (CRC) using isolated mitochondria from skeletal muscle. Significant main effects by Sod1 and mPRDX3, and interaction effect. *n* = 5–7. (d) Representative calcium tracings during CRC assay. Individual spikes represent calcium chloride, which was sequentially injected every 1 min until the opening of permeability transition pore (PTP) opening (inset). Values are shown as mean ± SEM. Data were analyzed using ordinary two‐way ANOVA with Tukey post hoc tests. *p < 0.05. GM, glutamate (10 mM) and Malate (2 mM); ADP (2 mM); Suc, succinate (10 mM); Ca, calcium (2 μM)

The rate of hydrogen peroxide generation in Sod1KO was elevated in skeletal muscle mitochondria in a basal state (State 1) without addition of exogenous substrates or inhibitors of the mitochondria electron transport system (Figure 2a), which is consistent with our previous reports (Jang et al., 2010, 2012; Muller et al., 2006). Increased mitochondrial hydrogen peroxides in Sod1KO, however, were abrogated by mPRDX3 upregulation (Figure 2a). Note that markers of oxidative modifications, determined by protein carbonyls and F_2_‐isoprostane, were unchanged by mPRDX3Tg (Figure S2). To assess electron transport system activity of the mitochondria (i.e., respiration), we determined oxygen consumption rate (OCR). Complex I‐ and II‐activated OXPHOS capacities were significantly decreased in Sod1KO, but the declines were protected by overexpression of mPRDX3 (Figure 2b). Another key function of mitochondria is to buffer cytosolic calcium ions, regulating the calcium concentration in cytoplasm. Thus, we measured mitochondrial calcium retention capacity (CRC) of isolated skeletal muscle mitochondria by challenging the mitochondria with sequential additions of calcium chloride (Figure 2d, inlet) until the opening of permeability transition pore. We showed significantly impaired mitochondrial calcium buffering capacity in Sod1KO mice consistent with our previous report (Jang et al., 2010), which is prevented by PRDX3 overexpression (Figure 2c,d). Note that our assessments to estimate mitochondrial contents, mitochondrial DNA copy number, or VDAC1, indicated no change in mitochondrial contents. The mRNA level of PGC1α was also unchanged by increased expression of PRDX3 (Figure S3). In summary, our data demonstrate that increased expression of mPRDX3 normalized the elevated mitochondrial hydrogen peroxide in Sod1KO muscle, and improved mitochondrial functions, including activity of the electron transport system and calcium buffering capacity.

**FIGURE 3:**
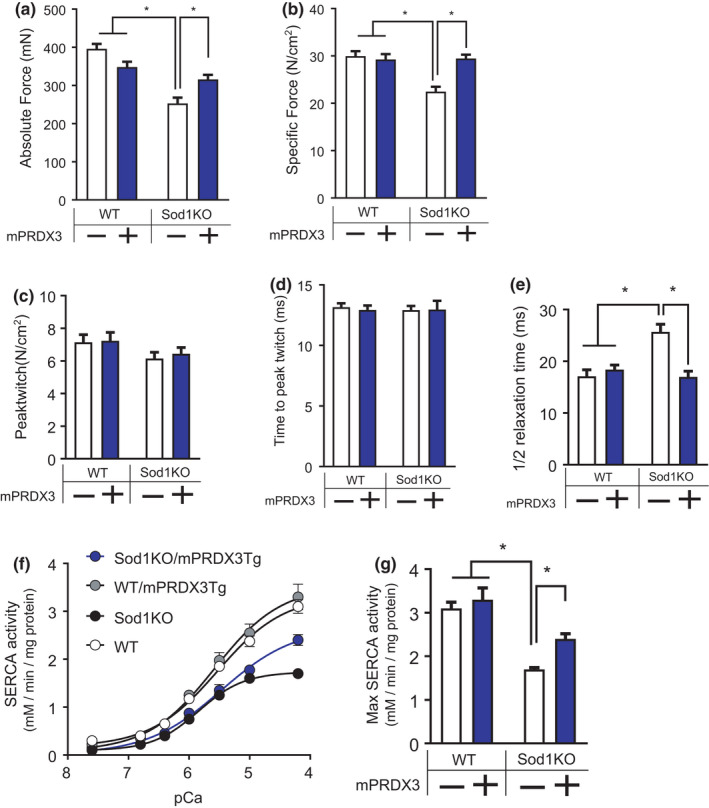
Contractile properties of skeletal muscle are impaired in Sod1KO mice, but protected by PRDX3 overexpression. (a) Maximum isometric force (mN). Significant main effect by Sod1 and interaction effect. *n* = 6–8. (b) Maximum isometric specific force, force per estimated cross‐sectional area (N/cm^2^). Significant main effect by Sod1 and PRDX3, and interaction effect. *n* = 6–8. (c) Peak twitch tension (N/cm^2^). *n* = 6–8. (d) Time to reach peak twitch (ms). *n* = 6–8. (e) Time to reach one‐half relaxation after twitch contraction (ms). Significant main effect by Sod1 and PRDX3, and interaction effect. *n* = 6–8. (f) SERCA activity plotted in response to increasing calcium concentration in gastrocnemius homogenates. *n* = 4. (g) Maximum SERCA ATPase activity. Significant main effect by Sod1 and PRDX3, and interaction effect. *n* = 4. Values are shown mean ± SEM. Data were analyzed using ordinary two‐way ANOVA with Tukey post hoc tests. *p < 0.05. SERCA, sarco/endoplasmic reticulum calcium ATPase

To determine the effects of mitochondrial hydrogen peroxide in muscle quality, we assessed *in vitro* contractile properties of isolated glycolytic muscle (i.e., EDL). Maximum isometric force was decreased by ~30% in Sod1KO mice, but the force deficit was partially rescued in Sod1KO/mPRDX3Tg mice (Figure 3a). Furthermore, maximum isometric specific force, force per cross‐sectional area (a measure of muscle quality), was fully restored by mPRDX3 overexpression (Figure 3b). We indirectly determined calcium kinetics of EDL muscle by analyzing twitch profiles. We found no change in twitch force or time to peak in Sod1KO (Figure 3c,d), but half relaxation time (1/2RT) was increased, suggesting impaired calcium reuptake mechanisms. 1/2RT was restored in Sod1KO/mPRDX3Tg mice (Figure 3e). To further assess cytoplasmic calcium kinetics in muscle, we determined the activity of the Sarco‐Endoplasmic Reticulum Calcium ATPase (SERCA) pump, which plays a critical role in calcium reuptake during muscle relaxation. Consistent with our previous reports (Xu et al., 2021), the SERCA activity was significantly impaired in Sod1KO mice, but was partially improved in the Sod1KO/mPRDX3Tg mice (Figure 3f,g). It is possible that calcium storage proteins are increased inside of SR, as recently demonstrated by a model of mitochondrial‐targeted catalase overexpression (Xu et al., 2021) or that PRDX3 overexpression can modulate the oxidation of specific residues in the SEARCA ATPase that are known to be inactivated by oxidation (Dremina et al., 2007). Increased mPRDX3 expression improves muscle quality, which are associated with enhanced calcium kinetics and handling in the cytoplasm.

**FIGURE 4:**
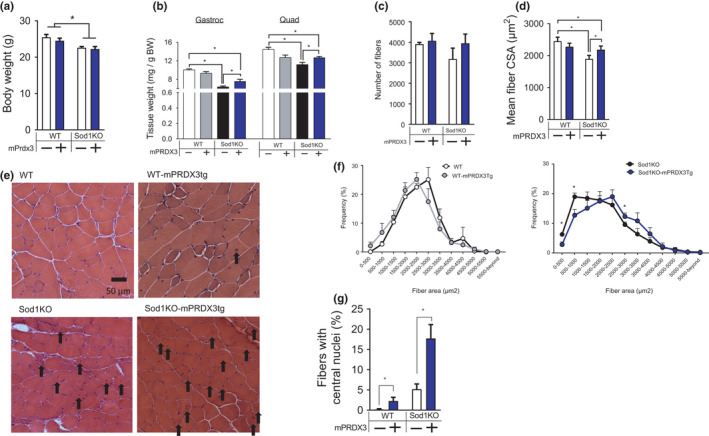
Sod1KO mice exhibit a significant reduction in muscle mass, which are partially protected by PRDX3 overexpression. (a) Body weights were significantly decreased in the Sod1KO and Sod1KO/mPRDX3Tg mice. Main effect by Sod1KO. *n* = 8–12. (b) Relative weights of gastrocnemius and quadriceps (mg muscle mass normalized by g body mass) were significantly reduced in the Sod1KO mice, but Sod1KO/mPRDX3Tg mice are protected. *n* = 8–13. (c) The number of fibers in gastrocnemius did not differ by Sod1 or mPRDX3. (d) Mean fiber cross‐sectional area was decreased in Sod1KO, but partially protected by mPRDX3 overexpression. Data were analyzed using ordinary two‐way ANOVA with Tukey post hoc tests. (e) Representative images of gastrocnemius cross‐sections stained by H & E. Arrows indicates centrally localized nuclei in myofibers. (f) Relative frequency distribution of fiber cross‐sectional area comparing WT and WT‐mPRDX3tg, and Sod1KO and Sod1KO/mPRDX3Tg. Frequencies are for WT and Sod1KO are shown separately for clarity. *n* = 4–5. (g) Fibers with central nuclei are shown in percent of total (%).4–5 sections were analyzed per mouse. 4–5 mice were analyzed per group. Values are shown mean ± SEM. Data were analyzed using ordinary two‐way ANOVA with Tukey post hoc tests. *p < 0.05. Gastroc, gastrocnemius; Quad, quadriceps

**FIGURE 5:**
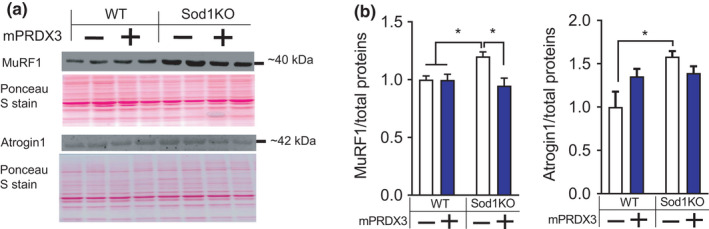
Increased expression of MuRF1 in Sod1KO is normalized by PRDx3 overexpression. (a) Immunoblot images showing MuRF1 and Atrogin1 expression. (b) Quantified immunoblot results of proteins *n* = 7–8. Values are shown mean ± SEM. Data were analyzed using ordinary two‐way ANOVA with Tukey post hoc tests. *p < 0.05

Sod1KO mice exhibited ~20% loss of body mass (Figure 4a). No changes in body composition were detected in this cohort, although previously we have noted reduced fat deposition in the Sod1KO mice (Muller et al., 2006). The mass of gastrocnemius and quadriceps was reduced by ~20–30% in Sod1KO mice compared with wild‐type mice, which was protected by mPRDX3 overexpression (Figure 4b). To further characterize the increase in muscle mass by mPRDX3tg, we performed a morphometric analysis to examine the number of fibers and fiber cross‐sectional area using muscle cross‐sections of gastrocnemius (Figure 4c,d). There was a significant decrease in fiber size in Sod1KO, which was protected by mPRDX3 overexpression (Figure 4d). Frequency distribution analysis of fibers reveals a rightward shift of the Sod1KO/mPRDX3Tg muscle compared with Sod1KO (Figure 4e,f). Our histological analysis also revealed increased number of fibers with central nuclei in wild‐type and in Sod1KO mice (Figure 4g). We found that expression of the atrogenes MuRF1 and atrogin1 were increased in Sod1KO, but MuRF1 protein expression was downregulated in the Sod1KO/mPRDX3Tg mice (Figure 5a,b). These data suggest that downregulation of protein degradation pathways is associated with the preservation of muscle mass by PRDX3 overexpression.

**FIGURE 6:**
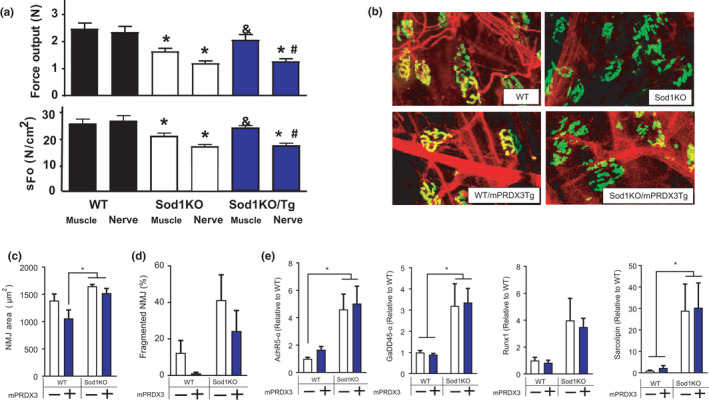
PRDX3 overexpression does not improve neuromuscular disruption in Sod1KO. (a) Maximum isometric absolute force (N) and specific force (N/cm^2^) either direct stimulation on muscle or through the sciatic nerve. * Significant difference compared with muscle‐stimulated force of WT. # Significant difference compared with nerve stimulation force of WT. & Significant difference compared with muscle‐stimulated force of Sod1KO. *n* = 6–8. (b) Representative neuromuscular junction (NMJ) immunofluorescence images from gastrocnemius muscle. Acetylcholine receptors (AchR) are pseudo‐colored in green and stained with Alexa‐488 conjugated α‐bungarotoxin. *n* = 3–5. (c) Quantification of NMJ area. Significant main effect by Sod1. (d) Percent of fragmented NMJs. *n* = 3–5. (e) mRNA levels of genes elevated in response to denervation and aging. Significant main effects by Sod1. *n* = 6. Values are shown mean ± SEM. Data were analyzed using ordinary two‐way ANOVA with Tukey post hoc tests. *p* < 0.05. NMJ, neuromuscular junction

Our laboratory has reported that skeletal muscle mtROS can induce NMJ impairment and contractile dysfunction (Ahn et al., 2019) and that mice lacking CuZnSOD have extensive NMJ disruption and weakness. To test whether downregulation of mitochondrial hydrogen peroxide in the Sod1KO mice can prevent NMJ disruption and muscle weakness, we determined the functional coupling between muscle and nerve by assessing contractile properties *in situ*. Maximum isometric specific force in Sod1KO is decreased by ~20% with electrical stimulation directly on the gastrocnemius, but the force deficit was ~40% when the muscle was stimulated through the nerve compared with WT (Figure 6a). These findings are consistent with a disruption of NMJs in Sod1KO skeletal muscle, in line with our previous reports (Larkin et al., 2011). Importantly, nerve stimulated force generation in Sod1KO/mPRDX3Tg mice was not improved by mPRDX3 overexpression, although the force generated by direct muscle stimulation was fully restored. These data suggest that the improvements in contractile properties were independent of neuromuscular impairment and are due to intrinsic changes in the muscle directly. To examine the neuromuscular junction morphology, we performed histological staining of motor neurons and NMJs and found no improvement in NMJ morphology phenotypes such as NMJ area or fragmentation in Sod1KO/mPRDX3Tg mice compared to Sod1KO (Figure 6b–d). Transcriptional markers of denervation, mRNA expressions of AchR‐α, GADD45‐α, Runx1, and Sarcolipin1, were elevated in Sod1KO consistent with our previous report (Sataranatarajan et al., 2015), and these remained elevated in the Sod1KO/mPRDX3Tg mice (Figure 6e). Collectively, our results demonstrate that scavenging hydrogen peroxides from skeletal muscle mitochondria improves muscle function independent of changes at the neuromuscular junction, that is, via a direct effect on the muscle tissue.

## 3 DISCUSSION

Our previous studies have clearly demonstrated that deletion of the *Sod1* gene results in high levels of oxidative stress that contribute to several pathological phenotypes, including accelerated loss of muscle mass and function associated with NMJ degeneration and elevated mitochondrial hydrogen peroxide generation. Here, we report that muscle‐specific overexpression of the hydrogen peroxide scavenger PRDX3 diminishes mitochondrial hydrogen peroxide release and prevents loss of mitochondrial electron transport chain activity and calcium retention capacity in Sod1KO muscle. Contractile dysfunction and muscle atrophy in Sod1KO are ameliorated by mPRDX3 overexpression, despite a lack of protection on NMJ disruption in Sod1KO mice. Overall, our results demonstrate that scavenging mitochondrial hydrogen peroxide prevents contractile dysfunction and attenuates muscle atrophy independent of protection against loss of NMJ structure and function in a redox‐dependent sarcopenia.

Impaired contractile properties in Sod1KO were rescued by mPRDX3 overexpression. This effect of Prdx3 is consistent with previous studies showing contractile and metabolic dysfunction in mice lacking Prdx3 (Lee et al., 2014; Zhang et al., 2016). It is reasonable to predict that at least part of the protective effect of PRDX3 on force generation in the Sod1KO/mPRDX3Tg mice is the prevention or reduction of oxidative damage or modification to contractile proteins, calcium regulatory proteins, or other critical proteins associated with contractile machinery due to increased scavenging of hydrogen peroxide from the mitochondria. Indeed, previous studies have demonstrated a dose‐dependent force reduction by excess hydrogen peroxide in isolated permeabilized single fibers (Plant et al., 2001), which was reversed by an antioxidant treatment dithiothreitol, DTT (Andrade et al., 1998). Fatigue‐dependent calcium insensitivity was also attenuated by DTT and mitochondria‐targeted antioxidant Tiron (Moopanar & Allen, 2005, 2006). Unexpectedly, we did not observe a global increase in markers of protein modification (i.e., carbonylation) in whole muscle tissue extracts from Sod1KO/PRDX3tg mice. The most direct explanation for this is that the increases in carbonyls in the muscle from the Sod1KO mice are not generated by elevated hydrogen peroxide, but rather other reactive oxygen species not scavenged by PRDX3.

Time to half relaxation after twitch tension (1/2RT) is increased in Sod1KO, but normalized in the Sod1KO/mPRDX3Tg mice. This could be associated with improved calcium buffering capacities as PRDX3 overexpression improved SERCA pump activity as well as mitochondrial calcium buffering capacity. In a similar model, Umanskaya et al. genetically overexpressed mitochondrial‐targeted catalase and demonstrated enhanced calcium release kinetics and increased SR calcium loads in muscle by scavenging mitochondrial hydrogen peroxide (Umanskaya et al., 2014). Calcium‐activated proteases are induced by high cytosolic calcium levels. It is possible that the improved calcium buffering capacity in the Sod1KO/PRDX3Tg mice might reduce induction of the activity of calcium‐dependent proteases, including calpain‐1 and caspase‐3 (Jang et al., 2010). Overall, impaired muscle quality in Sod1KO (i.e., specific force) was fully restored in the Sod1KO/mPRDX3Tg mice and may be at least in part associated with decreased hydrogen peroxide generation from the mitochondria and calcium kinetics within skeletal muscle.

Consistent with our previous reports in female Sod1KO mice at 8–10 months of age, the Sod1KO in this study exhibited ~20%–30% atrophy in the gastrocnemius and quadriceps compared to wild‐type mice (Ahn et al., 2019). The exact sequence of events leading to the loss of muscle mass and function in the Sod1KO mice is not completely defined, but our previous studies showed that NMJ disruption and loss of innervation are the key initiating event leading to increased mitochondrial hydrogen peroxide generation, activation of proteolytic pathways and reduced contractile function in both Sod1KO and old wild‐type mice (Jang et al., 2010). Elevated oxidative stress has been shown to cause protein modifications leading to increased susceptibility for ubiquitin‐proteasome system‐dependent degradation in skeletal muscle (Powers & Jackson, 2008). Indeed, Sod1KO mice show activation of cysteine proteases (i.e., calpain and caspase‐3), and increased activation of proteasome pathways (Jang et al., 2010). Myofiber atrophy in Sod1KO was reduced by mPRDX3 overexpression, while the number of fibers did not change. Notably, overexpression of mPRDX3 attenuated upregulation of the E3 ligase MuRF1 in the Sod1KO muscle. Our findings are consistent with a recent study showing that mice lacking MuRF1 are protected from muscle atrophy in cachexia induced by pulmonary hypertension (Nguyen et al., 2020). It is also possible that PRDX3 modulates signaling pathways of protein synthesis, or other proteolytic pathways involved in preservation of muscle mass and fiber CSA in Sod1KO, including autophagy pathways. The increase in the number of fibers with central nuclei in Sod1KO mice suggests muscle damage and regeneration consistent with what is seen in other injury (Morton et al., 2019) and disease models (Duddy et al., 2015). Of note, PRDX3 overexpression further increases the number of fibers with central nuclei. While the role of PRDX3 in central nucleation, injury, and fiber regeneration remains elusive at this point, it is possible that the antioxidant effects of elevated PRDX3 may enhance the regeneration response beyond that induced by fiber injury and denervation in Sod1KO mice. Increased regeneration has been proposed as a mechanism of skeletal muscle fiber hypertrophy in response to IGF1 overexpression and endurance exercise training (Paul & Rosenthal, 2002) and protection against ischemia‐induced muscle atrophy (Togliatto et al., 2013). Overall, the preservation of muscle mass in the Sod1KO/mPRDX3Tg mice was modest (Figure 3), suggesting that redox imbalance induced by excess hydrogen peroxide and its downstream pathways may not be the sole regulator of muscle mass in redox‐dependent sarcopenia, or that undermined effects of PRDX3 overexpression may have contributed to the moderate effect. The persistent NMJ disruption in the Sod1KO/mPRDX3Tg mice is also likely an important factor in the incomplete protection of muscle atrophy and the persistence of centrally nucleated fibers.

NMJ degeneration, retraction of motor neurons, and elevated muscle mitochondrial peroxide generation are common observations in aged humans (Oda, 1984; Tomlinson & Irving, 1977) and rodents (Chai et al., 2011; Ivannikov & Van Remmen, 2015), suggesting mitochondrial hydrogen peroxide as a driving factor in sarcopenia. Our laboratory demonstrated that elevated hydrogen peroxide and superoxide anion generation from skeletal muscle mitochondria in mice lacking mitochondrial superoxide dismutase (m*Sod2*KO) was sufficient to induce neuromuscular dysfunction and loss of force generation but not muscle atrophy (Ahn et al., 2019). To directly test skeletal muscle mitochondrial hydrogen peroxide as a therapeutic target, the present study targeted overexpression of the mitochondrial hydrogen peroxide scavenger PRDX3 to skeletal muscle in mice lacking CuZnSOD. Sod1KO mice are known to exhibit disruption of NMJ morphology, which remained impaired in the Sod1KO/PRDX3Tg along with increased markers of denervation. These data suggest that the improved sarcopenia phenotypes by PRDX3 overexpression are independent of NMJ impairment in our model of redox‐dependent sarcopenia. It is possible that other sources of ROS (i.e., NADPH oxidase or cPLA_2_) located in sarcolemma and/or bound lipid hydroperoxides, might be involved in NMJ impairment due to their spatial proximity (Pharaoh et al., 2020). Our group also investigated the role of mitochondria‐targeted catalase in skeletal muscle (mMCAT) in redox‐dependent sarcopenia. Contrary to our results in mPRDX3Tg, the mMCAT mice were fully protected from NMJ disruption as well as muscle atrophy and weakness in Sod1KO mice (Xu et al., 2021). The reasons for the discrepancy remain unclear, but one possibility would be the cofactor involvement for PRDX3, which is not required for catalase. Catalase is not present in the mammalian mitochondria, while PRDX3 scavenges presumably ~90% of the peroxides generated by mitochondrial ETS (Cox et al., 2009).

Our group previously demonstrated altered mitochondrial respiration and calcium buffering capacity in Sod1KO mice (Jang et al., 2012). Muscle‐specific PRDX3 overexpression not only reduces generation of mitochondrial hydrogen peroxide, but it also restores mitochondrial dysfunction elicited by redox imbalance. Although we did not challenge our mice on a treadmill, it is possible that the improved mitochondrial function may also contribute to increased exercise tolerance. This would be consistent with the results reported in a similar model expressing muscle‐specific mitochondrial‐targeted catalase in the Sod1KO mice. The mMCAT expression in the Sod1KO mice resulted in a significant increase in exercise tolerance (Xu et al., 2021). Improvements in mitochondrial functions by PRDX3 may or may not be related to an increase in mitochondrial contents. In order to estimate mitochondrial contents, we measured mitochondrial DNA copy numbers, and one of the proteins expressed in mitochondrial outer membranes VDAC1. There was no evidence of difference in mitochondrial contents in response to *Sod1* deletion or PRDX3 overexpression. The mechanisms by which PRDX3 improved mitochondrial function may be intrinsic without changes in mitochondrial content.

Skeletal muscle is comprised of slow and fast twitch muscle fibers. It has been widely accepted in the field that aging predominantly alters the fast twitch fibers in humans (Andersen, 2003; Lexell, 1995) and in mice (Murgia et al., 2017). Based on this, our study focused on muscles with predominantly fast twitch fibers. However, the idea of preferential effects of aging on fast twitch fibers has been challenged because myosin heavy chain co‐expressing (or hybrid) fibers dramatically increase with age, which is further complicated by misclassification of hybrid fibers (Purves‐Smith et al., 2014). A recent study shows that these hybrid fibers also undergo significant atrophy in elderly women (Sonjak et al., 2019). Others demonstrated similar effects across fiber types (Bodine & Baehr, 2014). Thus, we acknowledge that our current investigation is limited in glycolytic muscles, and it would be warranted to investigate other fiber types in the future. Also, we addressed the impact of scavenging hydrogen peroxide on neuromuscular disruption and mitochondrial function and redox status, but changes in redox balance will lead to broad impacts on skeletal muscle that are unexplored in the current investigation, including inflammation and satellite cell function.

In conclusion, our data demonstrate that increased expression of PRDX3 in skeletal muscle normalized the excess mitochondrial hydrogen peroxide generation in a redox‐dependent sarcopenia. This was associated with attenuated atrophy and rescue from contractile dysfunction, suggesting that mitochondria‐derived hydrogen peroxide plays a partial role in muscle atrophy but a larger role in impaired contractile function. We also report the novel finding that mPRDX3 overexpression improves muscle quantity and quality despite the lack of its impact on NMJ impairment in redox‐dependent sarcopenia. Thus, it would be important to determine if PRDX3 overexpression in muscle and neuron further protect muscle against atrophy and NMJ impairment in age‐associated sarcopenia. Overall, the results of our study indicate the significance of mitochondrial hydrogen peroxide and PRDX3 in sarcopenia, which can help narrow our targets for drug development of sarcopenia.

## 4 METHODS

### 4.1 Animal care

All mice were housed in pathogen‐free conditions and provided with water and food *ad libitum*. Adult (8–10 months old) female mice on a C57Bl6 background were used for this study. The Institutional Animal Care and Use Committee at Oklahoma Medical Research Foundation approved all procedures.

### 4.2 Mouse models

To evaluate the impact of mitochondrial hydrogen peroxide in sarcopenia, we used a mouse model with increased expression of a human PRDX3 transgene (PRDX3) specifically in skeletal muscle using a Cre‐Lox genetic approach (Figure 1a) as previously described. The construct contains a STOP codon flanked by LoxP sites upstream of the PRDX3 transgene. The STOP codon is released by Cre recombinase excision at the LoxP sites. The muscle‐specific PRDX3 mice were generated in a two‐step approach. First, female fl/fl mice were bred to male fl/fl mice that also carry a transgene for Cre recombinase driven by the human skeletal actin (HSA) promoter. PRDX3^fl/fl^ mice positive for HSACre^+/o^ constitutively express the PRDX3 transgene in skeletal muscle beginning early in embryonic development. For generation of mice with muscle‐specific overexpression of PRDX3 induced in Sod1KO mice, male PRDX3^fl/fl^xSod1^−/−^xHSACre^+/o^ mice were bred to female PRDX3^fl/fl^xSod1^+/−^xHSACre^+/o^ mice to generate PRDX3^fl/fl^xSod1^−/−^xHSACre^+/o^ (Sod1KO/mPRDX3Tg) and littermate Sod1^−/−^ mice negative for HSACre‐, PRDX3‐, or both (Sod1KO). The Sod1KO mice exhibit a redox‐dependent sarcopenia characterized by atrophy, contractile dysfunction, mitochondrial ROS, and NMJ disruption (Muller et al., 2006), which are phenotypes associated with sarcopenia in humans and animals. The genotype of the *Sod1* ^−/−^ mice was determined initially by standard PCR genotyping and confirmed by measuring the activity of CuZnSOD in a gel‐based assay using a method previously described and by immunoblot (Figure S1B).

### 4.3 Muscle functional assays

#### 4.3.1 In situ contractile properties

To isolate changes in NMJ function, we compared nerve versus direct muscle stimulation of contraction in an *in situ* contractile function preparation as we have previously described (Ahn et al., 2019). Briefly, gastrocnemius muscle was dissected from surrounding muscle and connective tissue using great care not to damage the neighboring nerves and/or blood vessels during the dissection. The hind limb was securely tied to a fixed post with 4‐0 monofilament nylon suture at the knee, and the foot was clamped to the platform. The distal tendon of the gastrocnemius muscle was then tied to the lever arm of a servomotor (Aurora Scientific). Muscle length was adjusted to the optimal length (*L*o) at which twitch force was maximal. With the muscle held at *L*o, 300‐ms trains of stimulus pulses were applied, which induced the maximum isometric tetanic force (Po). We stimulated the gastrocnemius muscle directly in order to bypass the neuromuscular junction and assess force generation.

#### 4.3.2 In vitro contractile properties

Contractile properties of extensor digitorum longus (EDL) were assessed *in vitro*. Mice were sacrificed using gaseous carbon dioxide, and EDL muscle was immediately excised and prepared for functional assays in a bicarbonate‐buffered solution gassed with a mixture of 95% O_2_ and 5% CO_2_ at room temperature. We placed the EDL muscle in an organ bath containing bicarbonate‐buffered solution at room temperature and determined the length that induces maximal twitch force, that is, optimal length (*L*_O_). Isometric force data were normalized by estimated muscle cross‐sectional area (CSA; N/cm^2^) for calculation of specific force. All data were recorded and analyzed using commercial software (DMC and DMA, Aurora Scientific).

### 4.4 NMJ imaging

We dissected fresh gastrocnemius muscle, cleaned connective tissue, and cut in small pieces in cold PBS. We fixed the tissue in 10% STUmol (Poly Scientific R&D, Bay Shore, NY) in ddH2O for 1 h on a rocker, washed three times for 5 min, and permeabilized in 2% Triton X‐100 in PBS for 30 min. After blocking overnight at 4°C in 5% NGS, 4% BSA, and 1% Triton X‐100 in PBS, the samples were incubated with primary antibodies for 24–48 h. The samples were washed six times for 30 min in PBS and incubated overnight with a secondary antibody and bungarotoxin conjugated to a fluorophore. The samples were washed again in PBS (six times for 30 min), blotted with a moistened Kimwipe, and mounted onto Superfrost Plus Slides (VWR) with EMS Glycerol Fluoromount with PPD anti‐fading agent mounting medium (EMS, Hatfield, PA), and coverslipped.

### 4.5 Mitochondrial isolation and functional assays

#### 4.5.1 Isolation

We isolated mitochondria from gastrocnemius muscle based on an established method in our laboratory. Gastrocnemius muscle was dissected, weighed, bathed in 150 mM KCl, and placed in Chappell‐Perry buffer, containing 100 mM KCl, 50 mM Tris, 5 mM MgCl_2_, 1 mM EDTA, and 1 mM ATP (pH 7.2), along with the protease from bovine pancreas (7.5 U/mL). The muscle was chopped using scissors and homogenized. The homogenate was centrifuged for 10 min at 600 *g*, with the supernatant then being passed through cheesecloth and centrifuged at 14,000 *g* for 10 min. The resultant pellet was washed once in modified Chappell‐Perry buffer, containing 100 mM KCl, 50 mM Tris, 1 mM MgCl_2_, 0.2 mM EDTA, and 1 mM ATP with 0.5% bovine serum albumin (BSA) and once in modified Chappell‐Perry buffer without BSA. Protein concentration was estimated using Bradford assay. Isolated mitochondria were used immediately following isolation for the functional assays described below.

#### 4.5.2 Rate of mitochondrial hydrogen peroxide generation

The rate of mitochondrial hydrogen peroxide production in isolated mitochondria was measured by Amplex Red (77.8 µM), horseradish peroxidase (HRP, 1 U/ml), and SOD (37.5 U/ml). The mitochondrial pellet was re‐suspended in a reaction buffer consisting of 125 mM KCl, 10 mM HEPES, 5 mM MgCl_2_, and 2 mM K_2_HPO_4_, pH 7.4. 40 μg of mitochondria per well was used for mitochondrial ROS measurements. All of the assays were performed at 37°C using black 96 well plates. Mitochondrial complex I was activated by glutamate (5 mM) and malate (5 mM), while complex II‐specific activation was achieved by succinate (10 mM) and rotenone (1 uM). Antimycin A (1 μM) was added to determine maximum rate of hydrogen peroxide generation. Fluorescence was followed at an excitation wavelength of 545 nm and emission wavelength of 590 nm. The slope of increase in fluorescence was converted to the rate of hydrogen peroxide generation using a standard curve.

#### 4.5.3 Oxygen consumption rate (OCR)

We added 20 µg of isolated mitochondria to each of the Oxygraph‐2K chambers (O2k, OROBOROS Instruments, Innsbruck, Austria). Rates of respiration were determined using sequential additions of substrates and inhibitors as follows: glutamate (10 mM), malate (2 mM), ADP (5 mM), succinate (10 mM), rotenone (1 µM), and Antimycin A (1 µM). All respiration measurements were normalized to Antimycin A to account for non‐mitochondrial oxygen consumption. Data were normalized by microgram of muscle mitochondrial proteins.

#### 4.5.4 Mitochondrial calcium retention capacity (CRC)

We challenged the mitochondria with a sequential addition of calcium chloride as we have previously described (Ahn et al., 2019). We used membrane‐impermeable calcium dye, Calcium Green‐5N, in O2k chambers. Mitochondria (100 µg) were injected in 2 ml of CRC buffer (in mM: 250 sucrose, 10 Tris, 10 KH_2_PO_4_, pH 7.4) containing substrates of mitochondrial complexes‐glutamate/malate (0.25 mM) and succinate (0.5 M). After 5 min of thermal equilibration, calcium chloride (1 µM) was added every 1 min until mitochondrial calcium release caused by permeability transition pore (PTP) opening. CRC was determined by the cumulative amount of calcium taken by the mitochondria.

### 4.6 SERCA pump activity

SERCA ATPase enzyme activity was measured in muscle homogenates at 37^○^C using a spectrophotometric assay. In brief, all muscle samples were homogenized following the ratio 1:10 with the SERCA homogenizing buffer, containing (in mM) 250 sucrose, 5 HEPES, 0.2 PMSF, 0.2% NaN_3_. After centrifugation of the homogenates, the supernatant was taken with the protein amount of 100 µg and mixed with the SERCA assay buffer containing (in mM) 200 KCl, 20 HEPES, 10 NaN_3_, 1 EGTA, 15 MgCl_2_, 5 ATP, 10 phosphoenolpyruvate, to generate a 3 ml mixture. Then, 18 U/ml of lactate dehydrogenase and pyruvate kinase, and 1 mM Ca^2+^ ionophore A‐23187 (C‐7522; Sigma) were added into the mixture. This reaction mixture was then aliquoted and mixed with CaCl_2_ to form eight different calcium concentrations with pCa points from 7.6 to 4.2 and a blank, and then loaded into a pre‐warmed 37°C quartz plate. The reaction was initiated by adding 1 mM NADH into the mixture, and the kinetic assay was done by the following settings: Temp = 37°C, Time = 30 min, *λ* = 340 nm, shaking between readings). The SERCA activity was calculated using the formula:

$$\mathrm{Total}\;\mathrm{ATPase}\;\mathrm{rate}=\frac{\mathrm{rate}\;\mathrm{of}\;A_{340\;\mathrm{nm}}\;\mathrm{signal}\;\mathrm{loss}\;\;\;}{\mathrm{pathlength}*6.23\;{\mathrm{mM}}^{‐1}{\mathrm{cm}}^{‐1}}$$

### 4.7 Marker of lipid peroxidation

The level of F_2_‐isoprostanes in gastrocnemius was determined by a previously described method (Roberts & Morrow, 2000). Briefly, 50–100 mg of muscle tissues were homogenized in 10 ml of ice‐cold Folch solution (CHCl_3_: MeOH, 2:1) containing butylated hydroxytoluene (BHT). The mixture was incubated at room temperature for 30 min. 2 ml of 0.9% NaCl was added and mixed well. The homogenate was centrifuged at 3,000 *g* for 5 min at 4°C. The aqueous layer was discarded while the organic layer was secured and evaporated to dryness under N_2_ at 37°C. After subsequent solid‐phase extraction and thin layer chromatography, F_2_‐isoprostanes were extracted and quantified by gas chromatography–mass spectrometry using the internal standard [^2^H_4_]8‐Iso‐PGF_2α_, which was added to the samples at the beginning of extraction to correct for yield of the extraction process. Esterified F_2_‐isoprostanes were measured using gas chromatography–mass spectrometry. The level of F_2_‐isoprostanes in muscle tissues was expressed as nanograms of 8‐Iso‐PGF_2α_, per gram of muscle mass.

### 4.8 Mass spectrometry‐based protein analysis

High‐resolution accurate mass spectrometry analysis was used to determine absolute concentrations of targeted proteins based on previously published methods (Ahn et al., 2019). The data were processed using Skyline version 3.7.0.10940. Protein abundance was determined by normalization to BSA used as a nonendogenous internal standard. Housekeeping proteins were also used for normalization.

### 4.9 Immunohistochemistry

Gastrocnemius muscles were frozen in liquid nitrogen‐cooled isopentane. 5 μm cross‐sections were made in the mid‐belly of the muscle using a microcryotome (−20°C) and dried for 1 h. The sections were rehydrated and stained with hematoxylin and eosin (H&E). H&E sections were scanned, and the images were used to determine cross‐sectional area and to count total number of myofibers in gastrocnemius muscles using Image J.

### 4.10 Immunoblotting

Tissue lysate and isolated mitochondria from gastrocnemius, as described above in mitochondrial isolation, were used to determine expressions of specific proteins. Table S2 provides information for the antibodies used for the study.

### 4.11 qRT‐PCR

Total RNA was extracted from gastrocnemius using TRIzol reagent (Invitrogen, Carlsbad, CA, United States). Equal amounts of extracted RNA (1 μg) were converted to first‐strand cDNA using a cDNA synthesis kit (Bio‐Rad, Herculus, CA, United States). 5 ng of the cDNA samples was amplified using primers for human PRDX3, mouse PRDX3, PGC1α, AchRα, GADD45α, Runx1, Sarcolipin, MuRF1, Atrogin1, Myogenin, and eMHC (details in Table S2) and SYBER green (Invitrogen, Carlsbad, CA, United States). Real‐time PCR (RT‐PCR) was performed in Quant Studio 6 (Applied Biosystems, Foster City, CA, United States). The ΔΔC_t_ method was used to calculate relative mRNA expression.

### 4.12 mtDNA copy number quantitation

mtDNA was absolutely quantified (copies per ng input DNA) by digital PCR as described previously (Masser et al., 2016) using a custom mtDNA fluorogenic primer‐probe copy number assay; Forward: 5′‐5′‐AGGACCTAAACTCAATAACGAAAGT‐3′; Reverse: 5′‐AGGTTTATGGC TAAGCATAGTGG‐3′; Probe: 5′‐/56‐FAM/ACACGACAG/ZEN/CTAAGACCCAAACTGG /3IABkFQ/‐3′ (IDT). This corresponds to mouse mitochondrial genome positions 423–525 (NC_005089.1). Genomic DNA was also absolutely quantified by the same digital PCR methods using a commercially available mouse TERT Reference assay (Thermo, # 4458368).

### 4.13 Statistical analyses

The results were analyzed using GraphPad Prism 9. 0. 1 (GraphPad Software, La Jolla, CA). For pairwise comparisons, unpaired two‐tailed *t*‐tests were used to compare means between groups. For multiple groups, ordinary one‐way ANOVA and two‐way ANOVA were performed with Tukey post hoc tests as indicated in the figure legends. Statistical significances were declared at *p* < 0.05. Data are presented as mean ± SEM.

## CONFLICT OF INTEREST

The authors declare that they have no conflict of interest.

## AUTHOR CONTRIBUTIONS

R.R. performed mitochondrial functional analyses and assisted B.A. with animal sacrifice. P.K. performed qRT‐PCR experiments and data analysis. H.X. performed SERCA pump assays and data analysis. K.P. performed experiments for neuromuscular junction morphology and data analysis. M.K. performed targeted mass spectrometry experiments and provided data for analysis. K.A.R., Q.R, and S.B. edited manuscript. H.V. designed the experiments and edited the manuscript. B.A. designed the experiments, coordinated data collection, performed muscle and mitochondrial functional assays, analyzed data, prepared figures, and wrote and edited the manuscript.

## Supporting information

Supplementary Material

## Data Availability

The data that support the findings of this study are available from the corresponding author upon reasonable request.
