# Supplementary material for: Scavenging mitochondrial hydrogen peroxide by peroxiredoxin 3 overexpression attenuates contractile dysfunction and muscle atrophy in a murine model of accelerated sarcopenia
Source: Aging Cell. 2022 Feb 24;21(3):e13569. doi: 10.1111/acel.13569 (PMC8920438; doi:10.1111/acel.13569)
Supplement: Supplementary file 1 — Supplementary Material [file ACEL-21-e13569-s001.pdf]

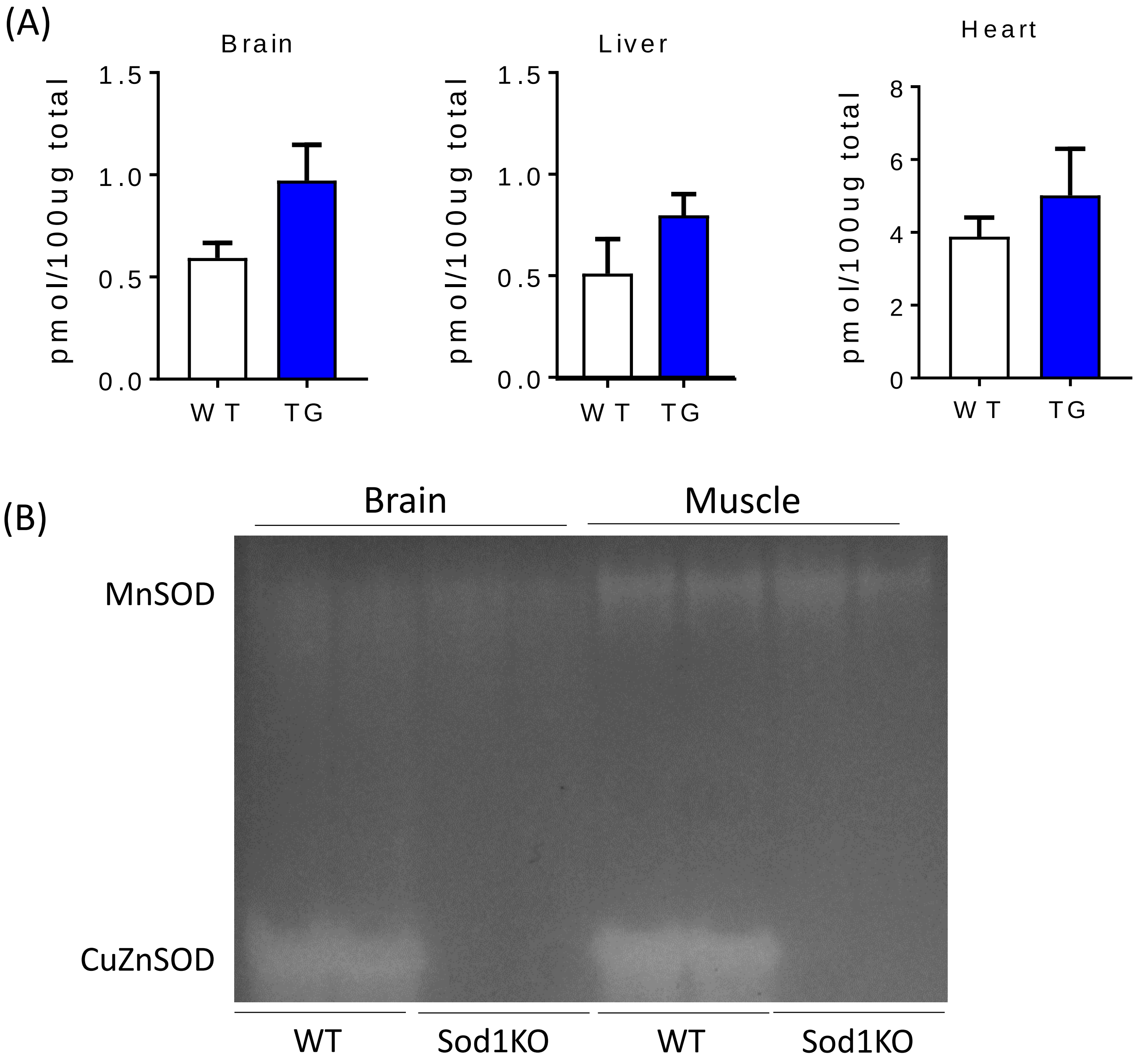
(A)
Brain
Liver
Heart
pmol/100ug total
1.5
1.0
0.5
0.0
8
6
4
2
0
WT
TG
(B)
Brain
Muscle
MnSOD
CuZnSOD
WT
Sod1KO
WT
Sod1KO

Figure S1

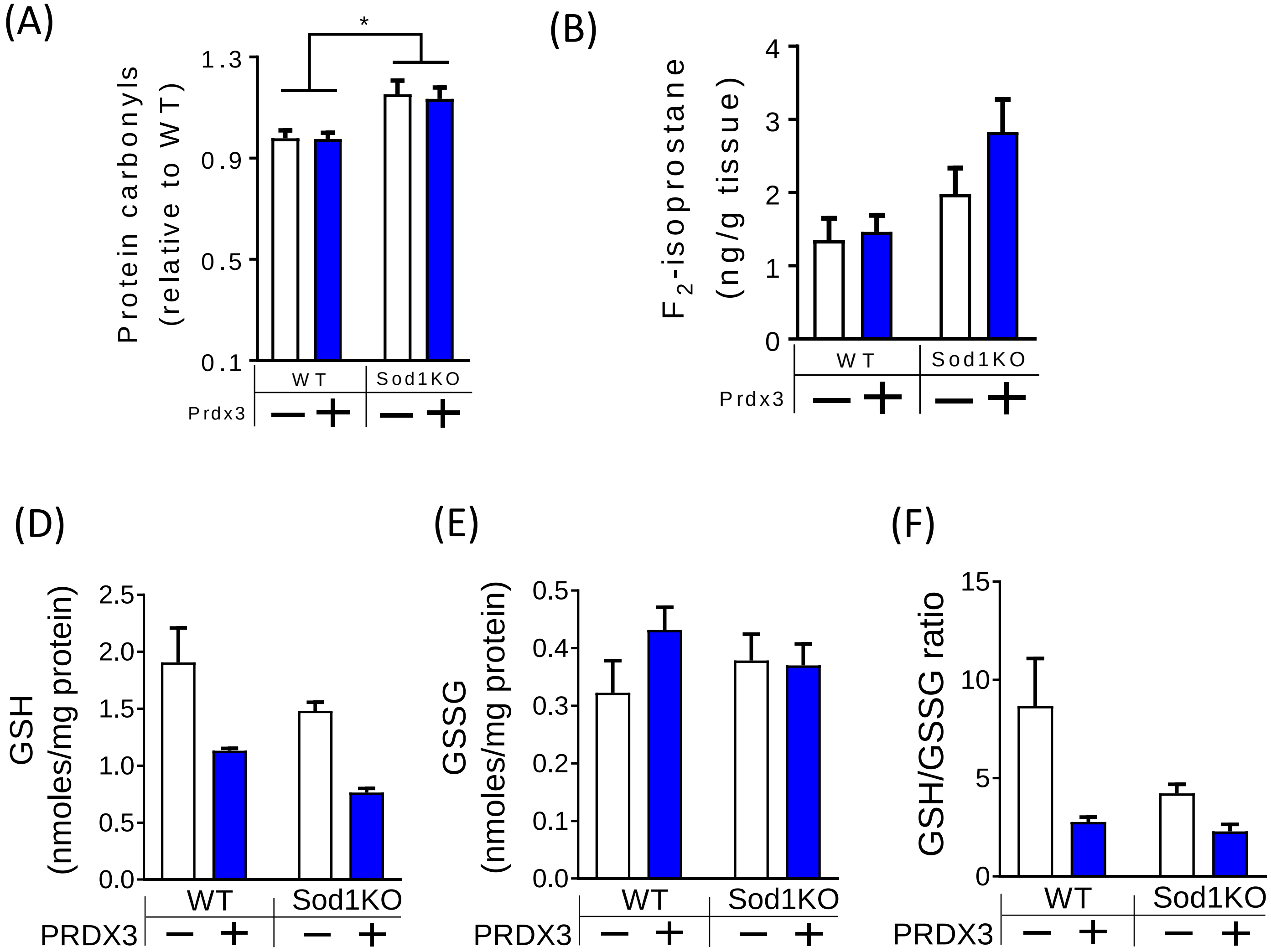
(A)
*
1.3
0.9
0.5
0.1
Protein carbonyls (relative to WT)
WT
Sod1KO
Prdx3
(B)
4
3
2
1
0
F_2-isoprostane (ng/g tissue)
WT
Sod1KO
Prdx3
(D)
2.5
2.0
1.5
1.0
0.5
0.0
GSH (nmoles/mg protein)
WT
Sod1KO
PRDX3
(E)
0.5
0.4
0.3
0.2
0.1
0.0
GSSG (nmoles/mg protein)
WT
Sod1KO
PRDX3
(F)
15
10
5
0
GSH/GSSG ratio
WT
Sod1KO
PRDX3

Figure S2

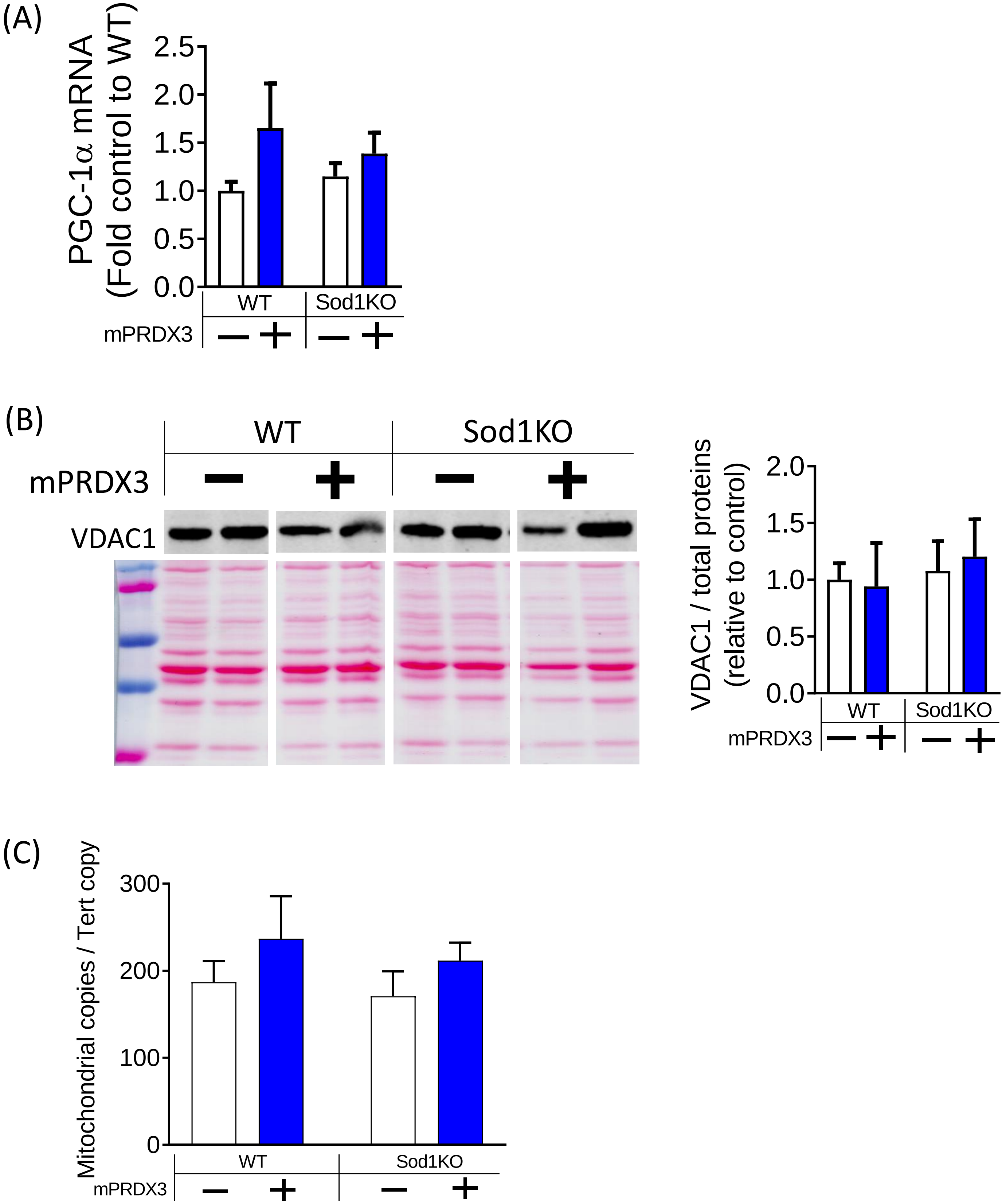
(A)
PGC-1α mRNA (Fold control to WT)
2.5
2.0
1.5
1.0
0.5
0.0
WT
Sod1KO
mPRDX3
(B)
WT
Sod1KO
mPRDX3
VDAC1
VDAC1 / total proteins (relative to control)
2.0
1.5
1.0
0.5
0.0
WT
Sod1KO
mPRDX3
(C)
Mitochondrial copies / Tert copy
300
200
100
0
WT
Sod1KO
mPRDX3

Figure S3

(A) Expression of proteins in muscle

Gene name	NCBI Gene ID	WT		WT-*Prdx3*Tg		*p-val*
Decr1	67460	0.33	±0.06	0.46	±0.07	0.047 *
Eno1	13806	0.24	±0.03	0.41	±0.05	0.014 *
Gpx4	625249	0.05	±0.01	0.06	±0.01	0.037 *
Gstm1	14862	2.23	±0.31	3.16	±0.32	0.040 *
HmgcL	15356	0.05	±0.01	0.07	±0.01	0.006 *
Hspa5	14828	0.13	±0.02	0.18	±0.01	0.004 *
Prdx3	11757	0.38	±0.05	1.43	±0.21	0.012 *
Slc25a20	57279	0.09	±0.02	0.14	±0.03	0.036 *

(B) Western Blots

Primary Antibodies	Company, Catalogue #	Ratio, incubation
Prdx3	Abcam, ab73349	1:1000, overnight
MuRF1	SCBT, sc-398608	1:500, overnight
MAFbx	SCBT, sc-166806	1:1000, overnight
Pax7	SCBT, sc-81975	1:1000, overnight
MyoD	SCBT, sc-377460	1:1000, overnight
Myogenin	SCBT, sc-52903	1:1000, overnight
Atkt-pan	CST, 4691	1:1000, overnight
pAkt-Thr	CST, 9275	1:1000, overnight
4EBP1	CST, 9644	1:1000, overnight

(C) qRT-PCR Primer Sequences

Gene Name	Forward Primer	Reverse Primer
hPrdx3	ACAGCCGTTGTCAATGGAGAG	ACGTCGTGAAATTCGTTAGCTT
mPrdx3	GCAGCTGCGGGAAGGTTGCT	TGCTGGGTGACAGCAGGGGT
PGC1a	CAGACCTGACACAACGCGGACAG	CCTGCGCAAGCTTCTCTGAGC
AchR-a	ACCTGGACCTATGACGGCTCT	AGTTACTCAGGTCGGGCTGGT
GADD45a	AGACCGAAAGGATGGACACG	GTACACGCCGACCGTAATG
Runx1	GATGGCACTCTGGTCACCG	GCCGCTCGGAAAAGGACAA
Sarcolipin	GAGGTGGAGAGACTGAGGTCCTTGG	GAAGCTCGGGGCACACAGCAG
MuRF1	ACCTGCTGGTGGAAAACATC	AGGAGCAAGTAGGCACCTCA
Atrogin1	GCAGAGAGTCGGCAAGTC	CAGGTCGGTGATCGTGAG
Myogenin	ACTCCCTTACGTCCATCGTG	CAGGACAGCCCACTTAAA
eMHC	GACTCAGCCGACACCATGAG	TTTTCCGACTTGCGGAGGAA

Table S
